# Lymphocyte Landscape after Chronic *Hepatitis C Virus* (HCV) Cure: The New Normal

**DOI:** 10.3390/ijms21207473

**Published:** 2020-10-10

**Authors:** Alip Ghosh, Sara Romani, Shyam Kottilil, Bhawna Poonia

**Affiliations:** Institute of Human Virology, University of Maryland School of Medicine, Baltimore, MD 21201, USA; alipghosh@ihv.umaryland.edu (A.G.); sromani@ihv.umaryland.edu (S.R.); skottilil@ihv.umaryland.edu (S.K.)

**Keywords:** HCV, DAA, immune recovery

## Abstract

Chronic HCV (CHC) infection is the only chronic viral infection for which curative treatments have been discovered. These direct acting antiviral (DAA) agents target specific steps in the viral replication cycle with remarkable efficacy and result in sustained virologic response (SVR) or cure in high (>95%) proportions of patients. These treatments became available 6–7 years ago and it is estimated that their real impact on HCV related morbidity, including outcomes such as cirrhosis and hepatocellular carcinoma (HCC), will not be known for the next decade or so. The immune system of a chronically infected patient is severely dysregulated and questions remain regarding the immune system’s capacity in limiting liver pathology in a cured individual. Another important consequence of impaired immunity in patients cleared of HCV with DAA will be the inability to generate protective immunity against possible re-infection, necessitating retreatments or developing a prophylactic vaccine. Thus, the impact of viral clearance on restoring immune homeostasis is being investigated by many groups. Among the important questions that need to be answered are how much the immune system normalizes with cure, how long after viral clearance this recalibration occurs, what are the consequences of persisting immune defects for protection from re-infection in vulnerable populations, and does viral clearance reduce liver pathology and the risk of developing hepatocellular carcinoma in individuals cured with these agents. Here, we review the recent literature that describes the defects present in various lymphocyte populations in a CHC patient and their status after viral clearance using DAA treatments.

## 1. Introduction

**Figure 1:**
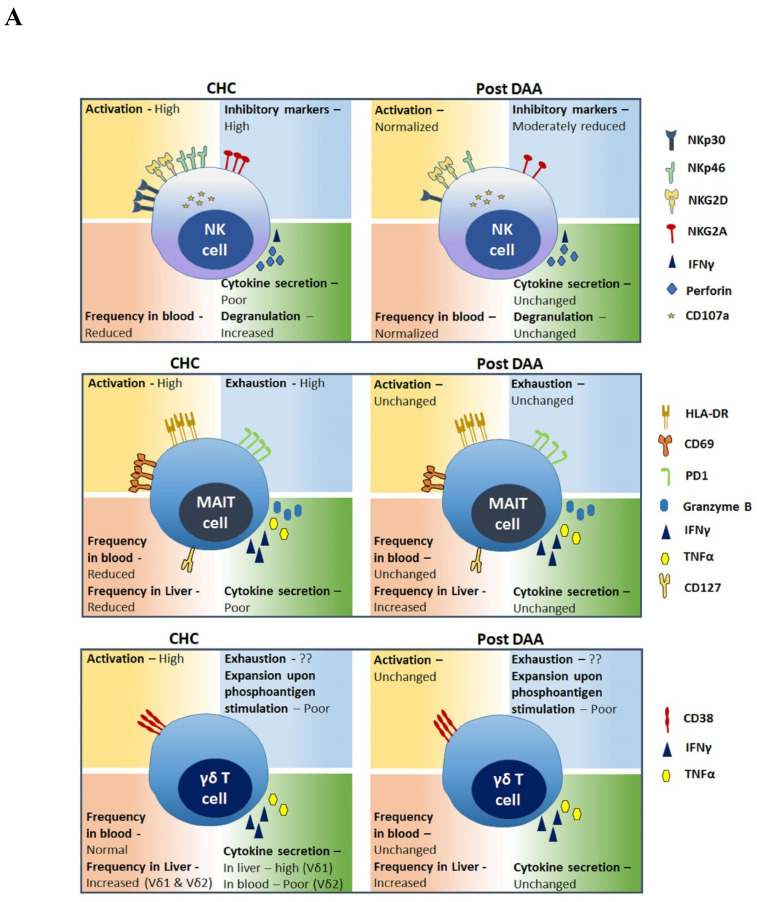

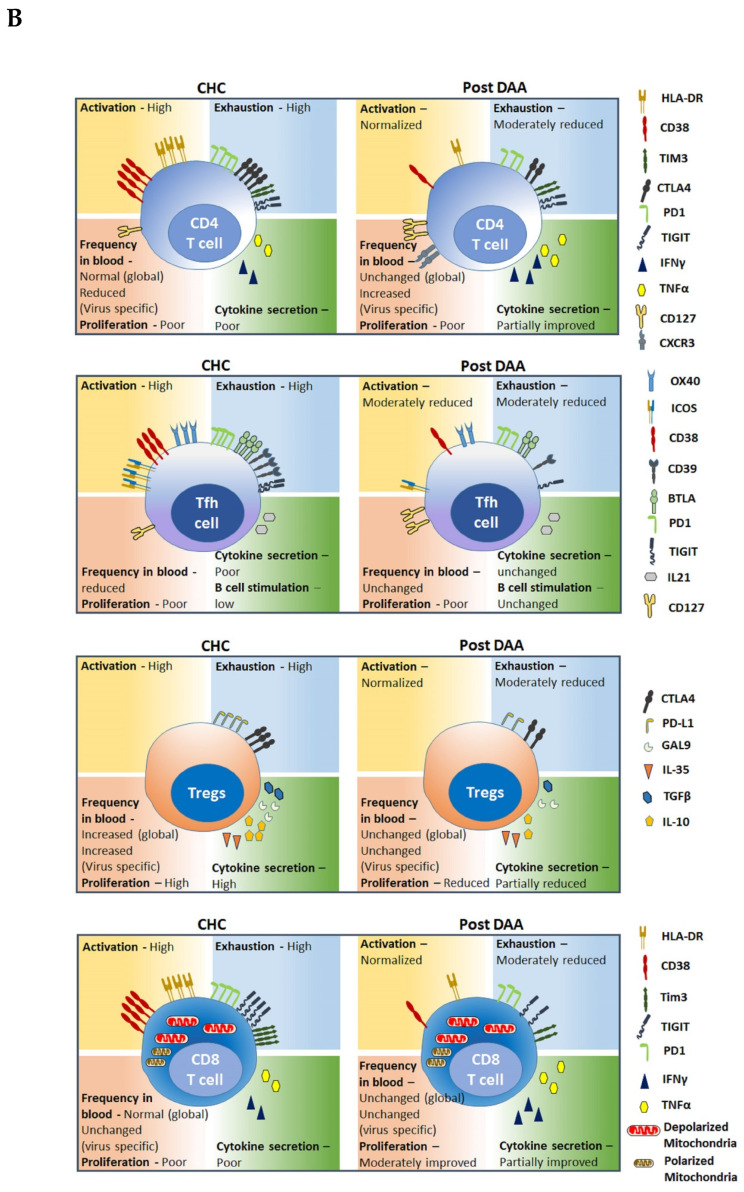
Visual summary of perturbations in frequency, phenotypes, and function of innate (**A**) and adaptive (**B**) lymphocytes in chronic *hepatitis C virus* (HCV) (CHC) patients and restoration associated with direct acting antiviral (DAA)-mediated viral clearance.

The ability to cure chronic *hepatitis C virus* (HCV) infection with direct acting antiviral (DAA) treatments [1] has provided scientists with a unique opportunity to investigate the immune recalibration that is possible once a chronic viral antigen has been removed. This is also an excellent model system to study basic immunology questions such as plasticity of the immune system and epigenetic regulation of immune responses in chronic infections and their reversibility. Our group has conducted pioneer clinical trials of DAA for HCV cure [2,3,4,5,6,7,8] and we investigate immune dysfunction in chronic HCV (CHC) and its recovery in cured individuals [9,10,11,12,13,14]. This focused review provides current understanding of innate and adaptive lymphocytes perturbations in CHC patients, the status of these immune cells after viral clearance with DAA, and implications thereof (summarized in Table 1 and in Figure 1).

## 2. Innate Immune Lymphocytes during and after Clearance of Chronic HCV Infection

Innate lymphoid cells are abundantly present in the liver [81,82], which is the site of HCV replication, and these are expected to provide the first line of defense against hepatotropic pathogens. A paradox common to most innate lymphoid cells is their function as early antiviral effector cells and their contribution to liver tissue injury by way of producing pro-inflammatory cytokines in response to pathogens.

### 2.1. Natural Killer (NK) Cells

Natural Killer (NK) cells are the dominant lymphocyte population in the liver and contribute to control of HCV infection as well perform anticancer effector function [83,84,85]. NK cells are a diverse population with multiple subtypes defined by unique combinations of surface markers. Traditionally, these cells are divided into types based on surface expression levels of CD16 and CD56 [83]. CD56^bright^CD16^dim^ NK cells produce antiviral cytokines [84], while CD56^dim/-^CD16^brigh^ NK cells express higher levels of killer immunoglobulin-like receptors (KIR) and perforin and are cytotoxic [83,85]. Additionally, activating and inhibitory NK cell receptors, NKG2D, NKp30, NKp46, and NKG2A, respectively, regulate NK cell functionality [86], and the balance shifts from inhibition to activation during HCV infection [15,16].

NK cells have been well investigated during and after cure of chronic HCV infection. The NK cell landscape is altered in the blood of chronically infected patients [17,18,19], reflected in increased frequencies of CD56^bright^ and decreased frequencies of CD56^dim^ subsets; DAA treatment-mediated HCV clearance results in an increase in CD56^dim^ and a decrease in CD56^bright^ NK cell frequencies, resulting in normalization [18]. Most of the other alterations in NK cell phenotypic markers observed in a chronically infected patient, including NKG2A, CD94, NKp30, NKp46, IFNγ, and perforin expression, also normalize rapidly upon virus clearance [19]. The majority of these changes are observed around week 12 or 24 after treatment with sofosbuvir and ledipasvir. In patients with undetectable viral loads treated with daclatasvir/asunaprevir, NKp46 and NKG2A expression levels normalized by week 8. The expression of inhibitory receptor NKG2A [19,20,21] and activating receptors NKp30 [18,19,22] and NKp46 [22,23] that increase on NK cells during HCV infection is also reduced with DAA treatment [18,19,22,24]. Expression of activating receptor NKG2D in chronic HCV infection remains unchanged during infection and with a cure in most studies [19,22,25,26] or was shown decreased during infection [27].

Functional impairment in NK cells during chronic infection is consistently reflected in reduced IFNγ production and higher CD107a degranulation response; these alterations return to normal responses upon virus clearance [17,22]. Whether improved NK cell functional responses upon virus clearance are indicative of generation of NK cell types which possess antigen-specific “memory” was investigated by Wijaya et al. [28]. This study showed that NK cells from patients have a stronger cytotoxic response against HCV infected cell lines compared with NK cells from healthy individuals, suggesting an antigen-specific response. Further, this cytotoxic ability was increased at 12 weeks after virus clearance. KLRG1 was identified as the marker of these antigen-specific NK cells; KLRG1^+^CD56^dim^ HCV-specific memory NK cells thus develop following HCV infection and may have relevance for HCV vaccine design.

A novel subset of “adaptive” NK cells representing a minor proportion of bulk NK cells, has been identified recently [29]. Compared with conventional NK cells, these CD57^+^ cells express lower levels of intracellular γ-signaling chain of Fc receptor (FcεRIγ), Siglec-7, NKG2A, NKp30, and Tim-3, higher levels of NKG2C and KIR, and possess higher ADCC activity. During HCV infection, an association of these adaptive NK cells with low levels of liver enzymes and fibrosis suggests a possible role of these cells in limiting fibrosis progression [87]. Phenotypically, the proportion of these cells is higher and they have increased PD-1 and reduced Siglec-7 expression in CHC patients with advanced fibrosis. A healthy individual’s adaptive NK subset is a greater IFNγ producer compared with its FcεRIγ+ conventional NK counterpart, and during chronic HCV infection, IFNγ production from adaptive NK is reduced. Upregulation of PD-1 expression during chronic infection is likely involved in negative regulation of this cell’s function, as evident from lower IFNγ and CD107a expression in PD-1+ compared with the PD-1- subset. Importantly, both the altered phenotype and function of these cells are restored with DAA-mediated virus clearance [29].

Strunz et al. [27] have comprehensively examined the NK cell repertoire using high-dimensional single cell analysis combined with novel metrics that capture the diversity and heterogeneity of these cells. This elegant work shows HCV infection results in increasing NK cell repertoire diversity in an individual, while inter-individual diversity in the repertoire decreases. In contrast with most studies however, no change in expression levels of multiple NK cell markers including CD38, NKp46, NKp30, NKG2D, and Programmed cell death protein 1 (PD-1) was observed during treatment and up to 36 weeks after DAA treatment. Some of the discrepancies described in different studies are potentially related to variables such as demographics of patient populations, regimen of DAA, viral genotype, and different methodologies, among others. For example, HCV genotype [20], the race of CHC patients [88,89], different DAA regimens [20], and non-response to pegylated interferon/ribavirin treatment [19] are all assumed to have an impact on the dynamic changes of NK cells during IFN free therapy.

Based on consensus, it can be concluded that most altered NK cell phenotypes and function observed in a chronically infected patient normalize with rapid clearance of HCV using DAA treatments.

### 2.2. Mucosal-Associated Invariant T (MAIT) Cells

Mucosal-associated invariant T (MAIT) cells are an innate-like T cell subset that comprise between 5 and 10% of peripheral T cells and about 12–50% of T cells in the liver and the gastrointestinal tract [30,31]. Human MAIT cells have a semi-invariant T cell receptor determining bacterial specificity, high expression of CD161, CD26, and IL-18Rα, and are able to produce cytokines including TNF-α, IFNγ, and IL-17 [30,31]. MAIT cells are readily activated by viral pathogens, including HCV and have a potential role in both host defense and immunopathology. During Chronic HCV infection, the frequency of MAIT cells decreases both in peripheral blood and liver [32,33,34,35]. In fact, MAIT cells were shown to be the most profoundly impacted immune cell type during CHC [32]. Intrahepatic MAIT cells are more activated and cytotoxic compared with their blood counterparts and frequencies of intrahepatic MAIT cells in CHC are inversely correlated to levels of liver inflammation and fibrosis [33]. This reflects the intrahepatic recruitment of MAIT cells in infected patients, where they become activated in the inflammatory cytokine environment and undergo apoptosis. MAIT cells are activated during CHC in a TCR-independent manner dependent on cytokines, predominantly IL-18 that is triggered by viral replication; these activated cells are believed to contribute to liver pathology [35]. The activation status of cells in periphery is reflected in increased expression of CD69, HLA-DR, and PD-1 and lower expression levels of CD127 [32]. Conversely, in vitro-activated MAIT cells have antiviral activity and limit HCV replication by IFNγ induction [35]. Thus, reduced frequencies of MAIT in HCV infection can limit their antiviral effect; at the same time, excess activation and cytokine production can contribute to liver pathology.

Hengst et al. demonstrated that interferon-free DAA therapy does not restore the peripheral MAIT cell compartment either phenotypically or functionally [32]. The frequency of peripheral MAIT cells remained at a low level in treated HCV-infected patients and these residual cells had high activation levels and low functional response. Significantly lower levels of IFNγ and TNF, less pronounced upregulation of CD69 and granzyme B, as well as lower degranulation in response to *E. coli* persist despite viral clearance. Similar results were reported by others for HCV [33,36] and for HCV/HIV patients treated with DAA [36,37]. The fate of intrahepatic MAIT cells, however, is different from peripheral MAIT cells after virus clearance. DAA treatment elevated the frequency of intrahepatic MAIT cells and resulted in their reduced activation and cytotoxicity [33]. On the other hand, it has been reported that IFNα-based therapies increase activation and frequency of MAIT cells in treated HCV patients [35].

Thus, clearance of chronic HCV infection with DAA therapy fails to recover the frequencies and function of this innate lymphocyte subset even long after cure. This is in contrast with the NK cell recovery that is both rapid and substantial. A consequence of persisting MAIT cell dysfunction is continued impaired bacterial pathogen immune surveillance in cured individuals.

### 2.3. Gamma Delta (γδ) T Cells

Gamma delta (γδ) T cells are a subgroup of T cells, expressing the TCR with γ and δ chains. Two main subsets of these cells are Vδ1 cells, present in gut and other epithelial tissues and Vδ2/Vγ9, the major γδ subset in the peripheral blood. γδ T cells are abundant in the liver, forming up to 5–15% of hepatic lymphocyte population in humans. In humans, a recent study described the CD27^lo^CD45RA^lo^ subset of Vδ1^+^ T cells expressing enhanced levels of liver residence-associated markers CD69, CXCR3, and CXCR6 in the liver [90].

Similar to other innate lymphocytes, both protective and pathogenic roles for these lymphoid cells have been suggested in the context of CHC. During HCV infection, a redistribution of γδ T cells to the liver is observed, and these cells express high levels of cytotoxicity markers and produce inflammatory cytokines IL-8, TNFα, and IFNγ [38]. An inflammatory subset of γδ T cells expressing CD161 was also found enriched in livers of patients with chronic HCV infection [39]. Agrati C et al. showed increased concentration of Vδ1 T cells in livers of HCV infected patients and these cells produced IFNγ, levels of which correlated with degree of necroinflammation, further suggesting a role of these cells in hepatic pathology [40]. At the same time, IFNγ produced by Vδ2 T cells resulted in a reduction in HCV replication in vitro in HuH7 infected cells [41]. These contrasting outcomes of role of IFNγ indicate different effects of cytokines produced by different gamma delta T cell subsets in HCV pathogenesis or controls and require further investigations. Even the same subset of γδ T cell can have dual function in protection and pathogenesis. Vδ2 T cells in the circulation of CHC patients are activated and express NK-like cytotoxic molecules including CD56 and CD16, and have high expression of cytolytic molecules, which correlate positively with serum ALT levels. At the same time, these cells had reduced IFNγ-producing capacity, suggesting impaired antiviral function of the Vδ2 T cell in CHC [42]. Intrahepatic Vδ2 T cells from CHC patients had exhausted phenotype and reduced IFNγ response; however, they still maintained antiviral capacity [43].

Not many studies have examined the fate of these cells post HCV cure. We showed that the circulating Vδ2 T cells in patients who were cured of HCV with DAA remain dysfunctional. Patient Vδ2 T cells were activated and had poor proliferative response to phosphoantigens [12] and these defects did not normalize with virus clearance. Another study reported a similar fate of these cells in HIV/HCV co-infected patients [44]. Clonal diversity of γδ T cells during and after HCV infection was examined by Ravens S et al. using mRNA-based next generation sequencing. Both Vγ9+ and Vγ9- cells from patients had comparably complex repertoires than healthy controls and DAA treatment did not alter this at least in 1 year follow up [45].

Impact of HCV clearance on Vδ2/Vγ9 cells can be summarized as persisting dysfunctional phenotype and functional state in a cured patient. An important consequence of remaining defects can manifest in long-term tumor surveillance by this innate effector subset.

## 3. Adaptive Immune Lymphocytes during and after Clearance of Chronic HCV Infection

The adaptive immune responses are crucial in determining the fate, either acute or chronic, of HCV infection. While about 30% of HCV-infected adult individuals are able to eliminate virus-infected cells via strong and sustained CD8^+^ and CD4^+^ T cell responses, the rest develop chronicity. Even though the adaptive immune system does not clear a persistent HCV infection and spontaneous resolutions are rare, immunity exerts some control over infection as evident from exacerbated liver disease in settings of immunosuppressive therapy or co-infection with HIV [91,92,93]. The inability of the immune system to clear a persistent infection is a result of exhaustion and dysfunction of the immune system. Global and antigen-specific T cells are severely dysfunctional in a chronically infected patient, characterized by expression of multiple exhaustion markers and poor HCV-specific cytokine production. With HCV cure now achievable in most treated patients, a significant question that remains is whether the dysregulated immunity is restored upon viral clearance to levels where it protects from re-infection. This is important for determining a need for developing a prophylactic vaccine, especially in high-risk populations. Development of chronicity after reinfection was observed in a chimpanzee model after successful clearance of HCV with DAA [94]. Several cases of reinfection with HCV have been reported in human patients who had previously cleared HCV with DAA therapy [95,96], most with high-risk behaviors. Understanding the adaptive immune restoration in a cured patient will allow drawing of conclusions regarding persisting immune dysfunction as a potential risk factor for re-infection. Additionally, alterations in global T cell populations have potential relevance for overall immune competence and protection against unrelated pathogens.

### 3.1. Helper T Cells

CD4^+^ T cells or T helper cells execute diverse tasks after differentiating into lineages such as Th1, Th2, Th17, Tregs, follicular T helper, etc. Although broadly directed HCV-specific CD4^+^ T cell response is observed at early stages of infection in both acute and chronic patients, persistent viremia is associated with early proliferative defects of these cells and subsequent rapid depletion of virus-specific response [46]. Difficulty in detecting antigen-specific T cells in peripheral blood from chronically infected patients is due both to the small frequency of these cells and possible migration of cells to the site of virus replication in the liver. Recent studies have investigated class II tetramer binding CD4^+^ T cells in chronic HCV infection and their fate after virus clearance. In an elegant study with samples from 44 patients tested at baseline, during and after DAA-mediated cure, Smits et al. [47] showed a significant increase in HCV tetramer binding CD4^+^ T cells within 2 weeks of therapy initiation. This likely reflects the egress of virus-specific cells from the liver in an IP-10 gradient-dependent mechanism. Analyses of global CD4^+^ T cells revealed that frequency of T cells expressing CXCR3, which is expressed on the vast majority of liver-infiltrating CD4^+^ T cells in chronic HCV infection [48], is increased in the peripheral blood 1 to 2 weeks after DAA therapy initiation, suggesting an early emigration of liver-infiltrating lymphocytes from liver to blood [49]. This is accompanied by a rapid decline in levels of serum IP-10 (CXCL10, the ligand for CXCR3) [50].

Concomitantly, there are dynamic changes in exhaustion and activation levels of these cells that tightly control their differentiation, proliferation, and effector function. T cell exhaustion is characterized by sustained expression of multiple inhibitory receptors on T cells, including PD-1, 2B4, Cytotoxic T-lymphocyte-associated protein 4 (CTLA-4), CD160, T cell immunoglobulin and mucin domain-containing protein 3 (TIM-3), and Killer Cell Lectin Like Receptor G1 (KLRG1) [51,52,53,69,70]. Expression of inhibitory receptors, PD-1 and CTLA-4, on global CD4^+^ T cells is almost universal in the early phase of the infection, regardless of the outcome. With spontaneous resolution of the infection, expression of these inhibitory receptors fades away. In contrast, persisting viremia continues to drive T cell activation, PD-1 and CTLA-4 expression, and blocks T cell differentiation until the virus-specific cells disappear from the circulation [52].

The impact of virus clearance on these exhaustion and activation markers on CD4+ T cells is well documented. The frequency of virus-specific CD4^+^ T cells expressing PD-1, BTLA, and T cell Ig and ITIM domain (TGIT) stays high during and after treatment, although intensity of PD-1 expression is reduced significantly [47]. Higher levels of TIGIT expression on HCV-specific CD4^+^ T cells observed during CHC remains persistently elevated at least until 24 weeks from the end of DAA treatment [53]. There is a however normalization of activation status of antigen-specific CD4^+^ T cells, as indicated by the significant reduction in CD38, ICOS, and OX40 expression [47].

Interestingly, an increase in expression of memory-associated markers CD127 and TCF-1 on antigen-specific cells occurs with viral clearance, this however is not accompanied by changes in cytokine expression patterns of these cells [47]. During CHC, a redistribution of memory CD4^+^ T cells at the expenses of naïve cells occurs, with memory T lymphocytes displaying an activated and exhaustive phenotype; these changes are only partially restored with virus clearance [54].

With respect to the global CD4^+^ T cell population, frequency of peripheral CD4^+^ T cells increases significantly with virus clearance [49,55] and a re-differentiation of the T lymphocyte memory compartment results in a higher effector memory population with increased Tbet expression [55]. The expression of various exhaustion markers on global CD4^+^ T cells is not uniformly impacted by viral clearance. While no change in PD-1 expression on CD4^+^ T cells with virus clearance occurs, expression of TIM-3 reduces on several innate and adaptive cells including CD4^+^ T, NK, and NK-like T cells [56]. There is no consensus regarding the expressions of activation markers HLA-DR and CD38 on the CD4^+^ T cells with HCV clearance. Both a decreased expression of HLA-DR and CD38 post DAA therapy [13,49] and no significant change in their expressions at 12 weeks after end of therapy [57], is reported by different investigators. Functionally, an increase in IFNγ, IL-17, and IL-22 producing global CD4^+^ and CD8^+^ T cells is observed after DAA therapy [57].

Although DAA treatments successfully clear the virus, the observations of re-infection in cured patients warrant investigations into prophylactic vaccination strategies. Adenoviral-vectored vaccines encoding non-structural proteins of HCV have shown multi-specific, high-magnitude, and durable CD4^+^ and CD8^+^ T cell responses in healthy volunteers; these responses resemble “protective memory” detected in patients who undergo spontaneous HCV resolution [97,98,99]. However, similar robust CD4^+^ T cell response is not present in immunized CHC patients, including in those treated with interferon/ribavirin [98,99] or DAA therapy [97]. Thus, viral clearance likely does not promote resurrection of exhausted memory CD4^+^ T cells, resulting in poor CD4^+^ T cell response with these vaccines. Unlike CD4^+^ T cells, vaccine-induced CD8^+^ T cell response was readily detectable in the majority of CHC patients treated with interferon (IFN)-based therapy [98] or in chimpanzees treated with DAA [94], to suppress viral load during vaccination. In spite of detectable CD8^+^ T cell response, these vaccination strategies failed to control HCV replication due to sequence variation of circulating virus, lack of help from CD4^+^ T cells, and expression of inhibitory receptors, particularly on virus-specific intrahepatic CD8^+^ T cells (discussed in details in [55]) [100].

Interestingly, several studies have indicated that many persistently HCV-infected pregnant women achieve substantial decline in HCV viral load after childbirth, which is associated with re-appearance of HCV-specific CD4^+^ T cells with an effector-memory phenotype at 3 months postpartum [101,102,103]. In addition, significantly increased frequencies of IL-2^+^, IFNγ^+^, and IL-2^+^IFNγ^+^ HCV-specific CD4^+^ T cells are detected at 3 months postpartum compared to the third trimester of pregnancy. However, PD-1 expression on HCV-specific CD4^+^ T cells remained high and correlated with viral control, which is indicative of its role in CD4^+^ T cell activation rather than exhaustion in these cases. In contrast, reduced CTLA-4 expression was associated with higher viral control, indicating its role as a negative regulator of HCV-specific CD4^+^ T cell activity in the postpartum period [103]. Such studies indicate that revival of HCV-specific CD4^+^ T cell immunity is possible after persistent infection and it can play a crucial role in controlling HCV replication.

PD-1 is one of the most well studied negative regulators of T cell response and its high expression level is an indicator of an exhausted immune response. We showed that in a cohort of patients that were treated for an ultra-short duration of 4 weeks with DAA, those that achieved SVR had higher levels of PD-1 and other “inhibitory receptor” co-expression on their CD4^+^ and CD8^+^ T cells before and at the end of treatment, although the levels of these markers significantly decreased 12 weeks after the end of treatment in the only SVR group [11]. Since these markers are expressed in response to continuous antigenic stimulation, it makes sense that their expression is high on virus-specific cells. We believe that the T cells co-expressing multiple inhibitory markers reflect antigen-specific cells and once the viral antigen level was reduced with 4 weeks of treatment, patients who had higher levels of such antigen-specific cells continued to control virus replication even when treatment was stopped at 4 weeks. This suggests that concentration of these markers reflects an activation of T cells in response to antigenic stimulation and not necessarily a terminally exhausted phenotype, which cannot be restored.

In summary, DAA therapy partially improves the CD4^+^ T cell compartment after HCV clearance. The frequencies of global CD4^+^ T cells are increased after DAA therapy and virus-specific CD4^+^ T cells transiently increase in circulation, possibly due to emergence from intrahepatic compartment. Expression of activation markers on antigen-specific CD4^+^ T cells is significantly reduced after virus clearance but expression of inhibitory receptors is reduced only moderately with modest improvement in cytokine production.

### 3.2. Follicular Helper T (Tfh) Cells

Humoral immune response requires interaction between antigen-specific B cells and CD4^+^ helper T cells. CD4^+^CXCR5^+^ follicular helper T cells (Tfh) cells are a specialized subset of CD4^+^ T cell that are essential for germinal center formation and production of high-affinity antibody by B cells in lymphoid follicles [104,105]. These cells express BCL-6 transcription factor after being primed and activated by dendritic cells and in turn, exhibit a unique CXCR5^hi^CCR7^lo^PD-1^+^ICOS^+^ phenotype [106,107,108,109]. Tfh cells promote proliferation, differentiation, and maturation of antigen-specific B cells inside lymphoid follicles through various cytokines, including the signature cytokine IL-21 [104,110,111].

Unlike acute HCV infection, where circulating virus-specific Tfh cells can be detected in periphery, these cells disappear from peripheral blood but remain detectable in the liver in CHC patients [48]. Comparing CHC and healthy donors, Spaan M et al. found no difference in the frequency of circulating CD4^+^CXCR5^+^ CXCR5^+^, CXCR5^+^PD-1^+^, CXCR5^+^ICOS^+^, and CXCR5^+^PD-1^+^ICOS^+^ subsets of CD4^+^ T cells between the groups [58]. In CHC patients with Cryoglobulinemia Vasculitis however, frequency of Tfh cells is higher compared to healthy donors and a reduction in Tfh frequency occurs after DAA therapy [59]. Although circulating Tfh cells in CHC patients express and secrete less IL-21 compared to healthy subjects, they are capable of stimulating memory B cells into IgG and IgM-producing plasmablasts [58,60]. Smits et al. demonstrated that the frequency of HCV-specific CD4^+^ T cells increased in circulation within two weeks of the initiation of DAA therapy [47] and the majority of these HCV-specific CD4^+^ T cells exhibited phenotypic and transcriptional characteristics of follicular T helper cells; these are maintained after therapy-induced elimination of persistent HCV infection. Following viral clearance with DAA, a downregulation in expression of exhaustion (PD1, BTLA, CD39, and TIGIT) and activation (CD38, ICOS, and OX40) markers and an upregulation in memory-associated markers (CD127) are detected on Tfh cells [47]. Thus, the antigen-specific Tfh reappear in circulation in a cured patient, and are maintained for months thereafter. Moreover, this reshaping of circulating HCV-specific Tfh is accompanied by reduced germinal center activity and preceded reduction on HCV-specific neutralizing antibody titers [47]. Whether these antigen-specific Tfh can induce long lasting protective antibody response will be an important consideration of HCV vaccine efforts.

A special subset of follicular CD4^+^ T cells, the T follicular regulatory (Tfr) cells, expresses regulatory markers CD25 and FoxP3 and functions to suppress Tfh cells, thus negatively regulating the germinal center [112,113,114,115]. The frequency of Tfr cells is increased both in the circulation and liver of a chronically infected patient [116,117]. This increase in intrahepatic Tfr cells could be a consequence of the immune modulatory effect exerted by HCV. Cobb, J.A. et al. demonstrated that HCV-infected hepatocyte-derived exosomes lead to an expansion of Tfr cells in a TGFβ-dependent manner [117]. However, it is not clear whether these Tfr cells are HCV-specific or how much role they play in suppression of HCV-specific Tfh. Virus clearance results in a reduction and normalization of their numbers with the concomitant increase in frequencies of Tfh cells [118].

### 3.3. T Regulatory Cells (Treg)

T regulatory cells (Treg) are CD4^+^ or CD8^+^ T cells engaged in promoting self-tolerance and suppressing non-self immune responses. Thus, they can play beneficial (prevent excess inflammation of the liver) or harmful (suppress anti-HCV immune responses) roles in the setting of chronic HCV infection. CD4^+^ Tregs, defined as CD4^+^CD25^+^CD127^low^Foxp3^+^ cells, are a specialized T cell population that inhibit the activation, proliferation, differentiation, and effector functions of multiple immune cell types, including T cells, B cells, NK cells, and dendritic cells [119,120,121,122]. Once activated, they exert their suppressive function in a non-specific manner through cell-to-cell contact inhibition and production of regulatory cytokines TGFβ, IL10, and IL35 [123]. In CHC, CD4^+^ Treg frequency gradually expands, resulting in reduced overall antiviral immune responses. A mechanism for increased Treg numbers during CHC is altered differentiation of CD4^+^ T cells in a persistently infected patient. While chronically infected patients often present with higher HCV-specific CD4^+^ T cell responses, both in terms of breadth and magnitude, this is largely comprised of regulatory T cells (CD4^+^CD25^high^CD134^+^CD39^+^). In comparison, HCV-specific CD4^+^ T cell responses in subjects who cleared the virus are comprised of effector T cells (CD4^+^CD25^high^CD134^+^CD39^−^) phenotype producing higher levels of IFNγ [61]. Galectin-9 (Gal-9), the ligand for TIM-3, is upregulated by Kupffer cells and monocytes and induces the expansion of CD4^+^ regulatory T cells (Treg) in a TGF-β-dependent manner in HCV-infected patients [62,63].

Abundant intrahepatic infiltration of Treg cells is present in CHC patients compared to healthy individuals [64,65], due to migration of these cells to liver. Monocyte-derived dendritic cells (mDCs) possess the capacity to generate Treg from naive CD4^+^ T cells in CHC patients and their interaction with HCV-infected hepatocytes results in increased production of the Treg-attracting chemokines CCL17 and CCL22, which facilitate intrahepatic recruitment of Tregs [124,125]. Whether Tregs limit or promote liver fibrosis is less clear [64,126,127]. Claassen et al. showed that activated Tregs migrate to the livers of CHC patients and limit the extent of HCV-induced immune activation, thereby limiting the extent of fibrosis [128]. Increased numbers of TGFβ producing HCV-specific Treg cells in chronic HCV-infected livers with limited fibrosis and normal serum ALT levels compared with CHC patients with elevated ALT levels support the role of this T cell in controlling liver pathology [64,129]. Langhans et al. however described a minor subset of intrahepatic IL-8 producing Tregs, present in close proximity to the areas of liver fibrosis, whose numbers correlated with stage of fibrosis; this subset also upregulated profibrogenic markers TIMP1, MMP2, TGF-beta1, alpha-SMA, collagen, and on hepatic stellate cells [126].

HCV clearance with DAA treatment does not affect percentages of Tregs in peripheral blood, even long-term after virus elimination [66,67,68]. Although the frequency of Tregs during DAA therapy gradually decreases until the end of therapy, in parallel with reduction in IL10 and TGFβ production, these changes are not sustained during the follow-up period, in spite of patient achieving SVR [67]. In spite of this, the Treg cells that reappear and expand after the end of DAA therapy might be functionally different from the Tregs present before therapy. Wei K et al. recently demonstrated that stimulation with Pam3Csk4, a TLR2 agonist, enhances the suppressive function and IL-35 production by T-regs that were isolated from a CHC patient before DAA therapy but not from Tregs isolated at SVR time-point. This indicates that therapy results in altered responsiveness of Tregs to TLR2 stimulation [66]. The fibrosis-limiting Tregs are retained in the liver up to at least 1 year after DAA-mediated cure [128], while the status of IL-8 producing fibrosis-promoting Tregs in the absence of HCV infection is unknown.

CD8 Tregs, defined as CD8^+^CD25^+^FoxP3^+^ cells, are a more recent discovery and are present at very low frequency in blood, making their characterization more difficult. One study has shown elevated frequency of CD8 Tregs both in the liver and peripheral blood of chronic patients, and this is associated with liver inflammation but not with fibrosis [130]. The status of this subset after HCV cure is not known.

The presence of Tregs in circulation long after the end of DAA therapy can impact the success of prophylactic vaccine candidates that are intended to prevent re-infection in cured individuals. In CHC patients, Han JW et al. evaluated the immunogenicity of an IFNL3-adjuvanted vaccine. Recombinant human IFNL3 reduced Treg cell frequency by inducing apoptotic cell death particularly of the activated Treg subpopulation (CD45RA^−^CD4^+^FoxP3^hi^); this was accompanied by an increase in virus-specific T cell responses [131]. Thus, innovative vaccination strategies that take into account the altered lymphocyte landscape in a cured patient might be critical for their success.

### 3.4. Th17 Cells

The Th17 subset of CD4^+^ T cells is distinguished by production of signature cytokines IL-17 and IL-22 and these cells play a key role in host resistance to microbial infections and to development of autoimmune diseases [132,133,134,135]. The transcription factors, orphan nuclear receptors ROR gamma t and ROR alpha (RORγt and RORα), determine the differentiation of CD4^+^ T cell to Th17 lineage with the influence of IL-6 and TGF-β [136,137,138]. Th17 are not well characterized in HCV infection. It is possible that these cells may have role in HCV-associated liver pathology as increased numbers of these cells correlate with levels of liver inflammation [139]. The Th17/IL-17 axis is believed to play a pro-fibrogenic role by way of its involvement in the production of cytokine IL-6 and profibrotic factors like TGFβ [140]. A skewing of intrahepatic T cells towards a higher Th17/FoxP3 ratio associates with advanced liver fibrosis in CHC and this is determined by TGFβ and TNFα concentrations in liver microenvironment [141]. On the other hand, IL-21 produced by Th17 cells can limit exhaustion of CD4 and CD8 T cells [142]. Different roles of this cell type in the context of both impact on antiviral effector function and in liver pathology have been explored. One report has examined the fate of these cells in DAA-cured patients so far. Comarmond C et al. showed increased frequencies of these cells in HCV patients with cryoglobulinemia vasculitis (CV) but not in those without CV. This Th17 polarization was normalized by end of treatment with DAA [59]. Thus, much remains to be known regarding their fate and significance in a cured patient.

### 3.5. Cytotoxic T Lymphocytes

Failure to eliminate HCV from infected hepatocytes is largely attributed to functional impairment of virus-specific CD8+ T cells, which is characterized by limited proliferative capacity, defective cytokine production and reduced cytolytic activity of these effector cells [71,72,73]. Two widely accepted mechanisms for the impairment of virus-specific CD8 T cells are the emergence of escape mutant variants of the wild type virus and CD8 T cell exhaustion [46,69,72,74,75]. Expression of Interleukin-7 (IL-7) receptor alpha chain (CD127), a key molecule associated with the maintenance of memory T cell populations, on CD8+ T cells is typically only observed when the respective antigen is controlled [143,144]. Cytokines, such as IL-7, and signaling through CD127 are critical for long-lived memory cells to survive and proliferate in the absence of antigen [145]. Whilst most resolved HCV patients express a high level of CD127 on more than 90% of the virus-specific CD8^+^ T cells [74], a substantial proportion (ranging from 10 to 100%) of the virus-specific CD8^+^ T cells express CD127 in long-established chronic HCV infection [74,76]. The high expression of CD127 in the virus-specific CD8 T cells in chronic infection is attributed to the emergence of escape mutant variant with mutation in the CD8^+^ T cell targeting epitopes due to immune selection pressure. In the absence of the targeting epitope, the effector CD8^+^ T cells re-differentiate into CD127 expressing memory-like CD8 T cells, while the HCV infection persists with mutant variants of viral antigens [74]. On the other hand, CD8^+^ T cells in CHC patients express a low level of CD127, generally accompanied by a higher level of activation marker CD38 and a lower level of CCR7 expression, insinuating re-activation-induced downregulation of CD127 on them [76]. The common feature observed in several chronic viral infections is exhaustion of T cells; persistent antigenic exposure of the virus-specific CD8 T cells to viral antigens leads to severe CD8 T cell exhaustion in chronic HCV-infected patients. Expression of multiple co-regulatory molecules such as PD-1, 2B4, TIM-3, Lag-3, CD5, CD160, and TIGIT is upregulated on HCV-specific CD8 T cells during chronic HCV infection [11,70], and is associated with poor proliferative and functional response. Varying levels of restoration of proliferation capacity of these virus-specific CD8^+^ T cells are observed following cessation of antigenic stimulation after DAA therapy-mediated viral clearance [77,78]. Factors that impact the proliferation capacity of the HCV-specific CD8 T cells upon peptide stimulation include the presence of cells recognizing the conserved epitopes [77], gender, presence of advance fibrosis [78], and combination of drugs used in the DAA regimen. Aregay A et al. showed a significant decrease in activation markers, CD38 and HLA-DR, on HCV-specific CD8^+^ T cells after DAA-mediated clearance of HCV and no significant reduction in the expression exhaustion markers, PD-1, TIM3, LAG3, and CD5 [78]. Likewise, the cytokine production from virus-specific CD8^+^ T cells was not restored after DAA therapy. However, a significant restoration of the proliferation capacity of these cells occurred in a subset of HCV patients that were treated with a combination of DAA drugs that included protease inhibitors but not sofosbuvir [78].

Whether expression of inhibitory molecules is indicative of persisting dysfunctional immunity in a cured individual is something not well understood. Chimpanzee studies showed that PD-1 is expressed on functional memory CD8+ T cells after resolution of infection and thus, is not always a marker of exhaustion [146]. We recently found that higher-level co-expression of PD-1 with other inhibitory receptors including CD160, 2B4, CTLA-4, Blimp-1, or Tim-3 on CD8^+^ T cells is present on HCV tetramer-positive cells and high expression of these molecules both at treatment start and at end of treatment time points correlated with the patients’ ability to clear chronic infection when treated with an ultra-short regimen involving 4 weeks of DAA treatment [11]. Importantly, expression of these molecules went down significantly in those who achieved SVR at week 16 but not in those that eventually relapsed. It is known from other viral infections that antigen-specific CD8^+^ T cells express PD-1, which preserves them from overstimulation, excessive proliferation, and terminal differentiation [147]. Thus, maintenance of PD-1 on T cells after HCV clearance potentially has a beneficial role in the preservation of these cells.

Recently, the role of high mobility group box (TOX) transcription factor as an important transcriptional regulator of exhausted CD8^+^ T cells in chronic viral infection was described [79,80]. Alfei et al. showed that TOX is highly expressed in HCV antigen-specific CD8^+^ T cells in CHC patients but not in those who spontaneously resolved HCV or in influenza-specific CD8^+^ T cells. Moreover, high expression of TOX in antigen-specific CD8+ T cells correlates with high PD1 expression in CHC patients [80]. In fact, TOX transcriptionally increases PD1 expression on antigen-specific CD8+ T cells in chronic infections, resulting in their reduced polyfunctionality [80]. After HCV clearance with DAA therapy, TOX expression is reduced in HCV-specific CD8^+^ T cells with concomitant reduction in PD1 expression [80]. Considering these data, targeting TOX could be a promising immunotherapeutic endeavor to reverse CD8^+^ T cell exhaustion and their disrupted polyfunctionality.

In an elegant work, Wolski et al. demonstrated differences in the transcriptional landscape of HCV-specific CD8^+^ T cells from patients during early acute HCV infection that either developed persistent infection or spontaneously resolved their infection [148]. This study identified molecular signatures of genes that govern the fate of virus-specific T cells towards memory or exhaustive phenotype; these signatures involved metabolic, nucleosomal, and immune processes. Among the differences, those who spontaneously resolved the infection showed higher levels expression of genes related to T cell differentiation and memory, while those who developed chronic infection had higher expression levels of genes related to T cell function, proliferation, and survival. Metabolic dysregulations at the transcriptional level involved genes related to nucleosomal regulation and those linked to T cell differentiation and the inflammatory environment. How clearance of HCV in these chronically infected subjects will reprogram these transcriptional signatures remains to be seen.

An emerging area in the field of T cell function is bioenergetic requirements of cells during a chronic viral infection [149]. Dynamic reprogramming of the metabolism occurs to meet excess requirements of T cell response under viral stress. Importantly, PD-1 along with mTOR signaling regulates these mitochondrial dynamics by repressing transcriptional coactivator PGC1a, leading to reduced cellular respiration, glycolysis, and dysregulated mitochondrial energetics [149]. Mitochondrial plasticity and bioenergetics are modulated significantly in chronic HBV and HCV infections [78,150]. Polarization status, superoxide level, and mass of the mitochondria dynamically influence survival, activation, and function of T lymphocytes [149,151,152,153,154]. Aregay A et al. elucidated the presence of mitochondrial dysfunction, characterized by depolarized mitochondria with increased mass and higher mitochondrial ROS in HCV-specific CD8 T cells, in CHC patients; these changes did not normalize following DAA-mediated clearance of HCV [78]. It remains to be investigated whether mitochondrial dysfunction is an effect of chronic infection or if it is a causal factor in impaired cellular function, exhaustion, and eventually, failure of antiviral T cells to control virus replication.

To summarize, DAA-mediated clearance of chronic HCV infection has partial impact on the recovery of CD8^+^ T cell dysfunction. While reduction in activation status and a partial reduction in the expression levels of exhaustion markers, specifically on antigen-specific cells, are evident, cytokine production, proliferation capacity and mitochondrial function of the CD8^+^ T cells remains dysfunctional.

### 3.6. B Cells

The role of B cells in HCV infection is not well understood. Neutralizing antibodies against the virus are present in all infected patients irrespective of outcome and it is not known if they play a significant role in virus control. Despite this, perturbations in B cell phenotypes are observed in chronically infected patients. The most common observation with respect to abnormality in B cell compartment is the presence of circulating mixed cryoglobulins in about 50% of patients, with overt cryoglobulinemia vasculitis (CV) in up to 10% [59]; these patients also have an accumulation of atypical memory (AtM) B cells (CD21^lo/-^ IgM^+^). In cohorts of patients with mixed CV that were treated with DAA, a decrease in percentages of these autoreactive B cells is reported, suggesting some recovery in B cell with virus clearance [59]. An extensive analysis of the mechanism behind Tbet^+^CD11c^+^CD27^+^CD21^−^ B cell-mediated autoimmunity showed the AtM derived antibodies do not react against ubiquitous autoantigens or HCV antigens, including NS3 and E2 proteins. These AtM-derived antibodies possess rheumatoid factor activity and target unique epitopes on the human IgG–Fc region. Virus clearance with DAA results in elimination of these deleterious B cells [155].

## 4. Liver Resident Lymphocytes in Chronic HCV Infection

While peripheral blood lymphocytes are recruited to tissues under conditions of viral infection, there are specialized populations of lymphocytes that reside in tissues that do not recirculate in the blood. These are called tissue resident cells and their role in chronic infections is being revealed now. Most peripheral lymphocytes have liver resident counterparts—memory CD8^+^ T (T_RM_) cells, invariant natural killer T (iNKT) cells, mucosal-associated invariant T (MAIT) cells, γδT cells, and innate lymphoid cells (ILCs) such as natural killer (NK) cells and other ILCs. These cells express phenotypic markers CD44, CD103, and CD49a, which mediate the adhesion and retention of these cells and possess a “memory-like” phenotype and function to provide long-lasting and robust protection against subsequent infection. For example, virus-specific CD8 T_RM_ can reside in the liver for a long time after primary infection and can control secondary infection with their cytolytic function [156]. On the other hand, these cells participate in liver pathology as well. Not much has been revealed about these cells during CHC yet. IL-2^high^T-bet^lo^Eomes^lo^Blimp-1^hi^Hobit^lo^ T cells were described enriched in livers from chronic HBV-infected patients who had partial immune control of the virus; these cells are believed to provide local surveillance [157]. The proportions of CD8^+^ T cells expressing CD103 was higher in CHC patients compared with healthy controls and these cells have an altered differentiation program indicated by lower levels of Hobit expression [158]. Further studies are required to understand the role of resident lymphocytes in HCV pathogenesis or control as well as their fate with and after chronic infection.

## 5. Summary

The human immune system efficiently controls most of the viral infections through strong sterilizing immunity mediated by various innate (non-specific; e.g., NK cells, gamma-delta T cells, MAIT cells) and adaptive (virus-specific; e.g., CD4^+^ Th1, Tfh, Th17 cells, CD8^+^ cytotoxic T cells, B cells) immune cell types. However, certain viruses including HCV can evade the immune system and develop chronic infection in some individuals. For development of chronicity, two fundamental events need to occur; first, the virus must evade the host sterilizing immunity and second, the host immune system must adjust to the continuous presence of viral antigen-driven inflammatory responses in order to limit viral replication to an acceptable level without permanent damage to infected tissues [159]. This adaptation comes in the form of transcriptional, metabolic, and epigenetic changes in immune cells. Whether these changes are permanent or reversible once the antigen is removed is a significant question in immunology, which can now be addressed in CHC patients that are cured with highly effective DAA treatments.

In this review, we discussed phenotypic and functional changes that occur in various lymphocyte populations during chronic HCV infection and their recovery upon clearance of HCV with DAA therapy. In our observation, NK cells, MAIT cells, CD4^+^ Th1 cells, and CD8^+^ T cells have been studied relatively extensively in comparison with other lymphocyte populations like γδ T cells, Th17 cells, Tfh cells, T regs, and B cells in the context of DAA-mediated clearance of HCV. In particular, studies on B cells and Tfh cells are scarce. Except NK cells, no other lymphocyte population undergoes significant recovery in up to one year follow-up studies after DAA therapy. A partial recovery manifested in reduced levels of activation and exhaustion markers and improved antigen-specific functional response is observed for CD4^+^ and CD8^+^ T lymphocytes. No significant recovery occurs in MAIT and γδ T cells. However, it is barely a decade since the implementation of these treatments and longer-term follow-up studies will provide more clear understanding of the reversibility of these immune defects.

## 6. Final Remarks

HCV clearance with DAA treatments has thus provided scientists with a first of its kind opportunity to investigate the extent of immune restoration that is possible once the antigen responsible for persistent immune dysfunction has been removed. Viral clearance has differential impact on different lymphocyte subsets, but overall viral clearance does not result in a “normalization” of lymphocyte landscape. Further studies can examine whether certain immune alterations take more time to normalize after cure or whether they are epigenetically imprinted by viral infection. The foremost significance of this is for a cured individual’s future immune competence, both in the context of re-exposure to HCV and for risk of associated pathology-like fibrosis and HCC.

## Figures and Tables

**Table 1:** Lymphocyte landscape present in a chronic *hepatitis C virus* (HCV)-infected patient and its status after virus clearance using direct acting antiviral (DAA) therapy.

Lymphocyte Populations	Dysfunction in CHC			Post DAA Therapy			Reference
	Frequency	Phenotype	Function	Frequency	Phenotype	Function	
**Natural Killer (NK) cells**	Reduced in blood	NKG2A↑, NKp30↑, NKp46↑	Reduced cytotoxicity, INFγ↓, CD107a ↑	Increase in CD56^dim^ and decrease in CD56^bright^	NKG2A↓, NKp30↓, NKp46↓, CD16↑, KLRG1↑, CD69↑	Improved cytotoxicity, INFγ↑	[15,16,17,18,19,20,21,22,23,24,25,26,27,28,29]
Mucosal-associated invariant T (MAIT) cells	Low in blood and liver	CD69↑, PD-1↑, HLA-DR↑, CD127↓	Increased cytotoxicity, INFγ↓, INFα↓, GrB↑	Low in circulation, Increase in liver	CD69↑, PD1↑, HLA-DR↑, CD127↓	Increased cytotoxicity, INFγ↓, INFα↓, GrB↑	[30,31,32,33,34,35,36,37]
Gamma detes (γδ) T cells							
Vδ1	Increased in liver	CD161↑	Increased cytotoxicity, INFγ↑, INFα↑,	ND	ND	ND	[38,39,40]
Vδ2	Unchanged in blood, Increased in liver	CD56↑, CD16↑, PD-1↑	Poor proliferation in response to phospho-antigen stimulation, INFγ ↓	Unchanged ih blood	ND	Poor proliferation in response to phospho-antigen stimulation, INFγ↓	[12,41,42,43,44,45]
Helper T Cells	Virus-specific CD4^+^ T cell reduced in blood	CD38↑, HLA-DR↑, PD-1↑, CTLA-4↑, TIM-3↑, KLRG1↑, TIGIT↑, CD127↓	INFγ↓, INFα↓	Virus-specific CD4^+^ T cell increased in blood	CD38↓, HLA-DR↓, PD-1↑, CTLA-4↓, TIM-3↓, TCF1↑, TIGIT↑, CD127↑, CXCR3↑	INFγ↓, INFα↓, IL-22↑, IL-17↑	[46,47,48,49,50,51,52,53,54,55,56,57]
Follicular Helper T (Tfh) Cells	Reduced in blood	CD38↑, CD39↑, ICOS ↑, OX40↑, BTLA↑, TIGIT↑, CD127↓	IL-21↓	Reduced in blood	CD38↓, CD39↓, ICOS↓, OX40↓, BTLA↓, TIGIT↑, CD127↑	IL-21↓	[48,58,59,60]
T Regulatory Cells (Treg)	Increased in blood and liver	PD-1↑, CTLA-4↑,	IL-10↑, GAL9↑, IL-35↑, TGFβ↑	Increased in blood	PD-1↑, CTLA-4↑,	IL-10↓, GAL9↓, IL-35↓, TGFβ↓ During therapy	[61,62,63,64,65,66,67,68]
Cytotoxic T Lymphocytes	Unchanged in blood	CD38↑, HLA-DR↑, PD-1↑, LAG-3↑, TIM-3↑, CD5↑, CD160↑, TIGIT↑, CD127↓, TOX↑	Reduced cytotoxicity, INFγ↓, INFα↓	Unchanged in blood	CD38↓, HLA-DR↓, PD-1↑, LAG-3 ↑, TIM-3↑, CD5↑, CD160↑, TIGIT↑, CD127↓, TOX↓	INFγ ↑, INFα ↑	[11,69,70,71,72,73,74,75,76,77,78,79,80]

ND: Not defined.
